# A Systematic Review of Severe Neurological Manifestations in Pediatric Patients with Coexisting SARS-CoV-2 Infection

**DOI:** 10.3390/neurolint13030041

**Published:** 2021-08-17

**Authors:** Lauren O’Loughlin, Nilo Alvarez Toledo, Leon Budrie, Randall Waechter, Joanna Rayner

**Affiliations:** 1 Department of Physiology, Neuroscience and Behavioral Sciences, School of Medicine, St. George’s University, St. George, Grenada; loloughl@sgu.edu (L.O.); RWaechte@sgu.edu (R.W.); 2 Department of Microbiology, Immunology and Pharmacology, School of Medicine, St. George’s University, St. George, Grenada; lbudrie1@sgu.edu (L.B.); jrayner@sgu.edu (J.R.); 3 Windward Islands Research and Education Foundation, St. George’s University, St. George, Grenada

**Keywords:** COVID-19, coronaviruses, neurological symptoms, pediatrics, encephalitis, anosmia

## Abstract

SARS-CoV-2 infection in children produces mild respiratory symptoms or no symptoms at all in most cases. Some pediatric patients develop a severe complication associated with high mortality, the multisystem inflammatory syndrome in children (MIS-C). In both scenarios, there are reports of neurological manifestations. This article aims to review the cases of pediatric patients with severe neurological issues and a coexisting positive SARS-CoV-2 test. A literature search was performed between March 2020 and May 2021. The results included the data from 41 studies, with 159 children with severe neurological manifestations, within an age range from 24 h to 17 years. The neurological disorders included 38 cases with stroke, 32 with encephalitis, 22 with encephalopathy, and 10 with Guillain–Barre syndrome. Sixty-five out of 159 cases with severe neurological manifestations were diagnosed with MIS-C. Direct neuroinvasion and the exaggerated immune response in some patients seem to be the most critical factors triggering these manifestations. Further research in the ongoing pandemic is needed to elucidate the precise mechanism.

## 1. Background

Severe acute respiratory syndrome coronavirus 2 (SARS-CoV-2) was first identified in Wuhan, China, in December 2019 and has become a global public health emergency [1]. The Johns Hopkins University developed an online coronavirus tracker that tracks cases, deaths, and recoveries worldwide. As of May 14 May 2021, over 160,000,000 cases and over 3,000,000 deaths in 192 countries have been reported (https://coronavirus.jhu.edu/us-map (accessed on 14 May 2021)) [2].

The disease has been devastating worldwide, causing social, health, and economic crises, the effects of which will be felt for decades to come. One of the fascinating features of SARS-CoV-2 is the broad spectrum of symptoms and complications seen in patients of all ages, races, and health statuses. In a clinical retrospective study from Wuhan, the most common symptoms of infection were fever (88.7%), cough (67.8%), myalgia, and fatigue (50%), as well as more uncharacteristic initial presenting symptoms such as anosmia and ageusia [3]. 

Some patients have experienced neurological symptoms, and evidence of neural infection has been seen in human and mouse brains [4,5,6]. This fact has lead to ongoing investigations about the mechanism of the neuroinvasive potential of SARS-CoV-2. The virus has an affinity for angiotensin-converting enzyme 2 (ACE2) receptors normally located on the human epithelial cells of the respiratory tract. However, there is evidence that ACE2 receptors can also play a role in the infection of the central nervous system, which explains the neurological symptoms reported in many patients. Associated histological findings based on mouse models indicate spike proteins in neurons, disruptions in the typical vascular topology expected in the cortex, and an important loss of the normal enrichment in radially oriented vessels in upper layers and associated cell death. There have also been reports of SARS-CoV-2 in CSF samples [6]. 

Most of the existing data about the neurological impact of SARS-CoV-2 come from case studies of individual patients with coexisting SARS-CoV-2 and neurological symptoms. Children are less likely than adults to experience symptoms when infected with the SARS-CoV-2 virus [7,8], and thus, current information from the pediatric populations mainly relies on case studies from individual patients. Like adults, the affinity of the virus to the ACE2 receptor and subsequent disruption to normal cellular functioning, immunologically mediated cytokine storms, and increased coagulation factors have been postulated as being responsible for the neurological findings in children [9,10]. A broad spectrum of neurological manifestations has been reported in pediatric patients, ranging from specific to nonspecific and from self-limiting to fatal. Cases of loss of smell and taste, headache, stroke, dizziness, seizures, and ataxia have been reported [9]. One review article on neurologic and radiologic findings in pediatric COVID-19-positive patients said that of 27 patients, four had neurological symptoms [10]. The most commonly reported results were encephalopathy, headache, weakness, and brainstem signs. 

The majority of existing review articles and case studies are focused on the adult patient population. Neurological symptoms, however, have been reported in both adult and pediatric patients. The aim of this article is to provide an updated review of the current published data on pediatric patients with COVID-19 and coexisting severe neurological manifestations. This information can inform healthcare providers about the major neurological issues they are most likely to see among the pediatric population with SARS-CoV-2. 

## 2. Methods

A systematic review was conducted according to the Preferred Reporting Items for Systematic Reviews and Meta-Analysis (PRISMA) statement and the PRISMA checklist and flow diagram [11]. 

### 2.1. Literature Search

A comprehensive literature search was performed using the PubMed, Hinari, Google Scholar, and Cochrane databases. References were identified from March 2020 to May 2021. The keywords “COVID-19”, “coronavirus”, “SARS-CoV-2”, and “COVID 19” were used in all the searches. The terms “child”, “children”, and “pediatric” were also used in every search. Associated terms such as “severe neurological disease” and “life-threatening neurological manifestation”, were then added. Boolean operators were used to link the different terms.

The research articles that emerged were limited to mostly case reports and case studies, given the specificity of the topic. No restrictions on the publication date of the articles were imposed.

### 2.2. Eligibility Criteria

The review included any study reporting major neurological manifestations, in laboratory-confirmed COVID-19 patients, within the age range of 0 to 21 years old (pediatric population) [12], in the English language. The review excluded papers reporting neurological symptoms in adult patients and neurological symptoms in patients with Middle East respiratory syndrome coronavirus (MERS-CoV) and severe acute respiratory syndrome coronavirus (SARS-CoV). The study also excluded patients that only presented with minor neurological manifestations such as headache, fatigue, and/or smell and taste dysfunction. 

### 2.3. Data Extraction

All the studies were screened, and data were manually extracted (N.A.T., L.O., L.B.). The variables used to describe the studies included the first author, type of design, site of the study, date of publication, and published journal. The variables used to describe the data of the patients included age, sex, neurological symptoms, and outcomes. 

### 2.4. Outcome Measures

The outcome of the present study was to elucidate and rank order the neurological manifestations of COVID-19 in the pediatric population reported in the medical literature. The details of the studies were described in the Table 1. The results were divided into two major categories: central nervous system manifestations and peripheral nervous system manifestations.

## 3. Results 

### 3.1. Study Characteristics 

**Figure 1:**
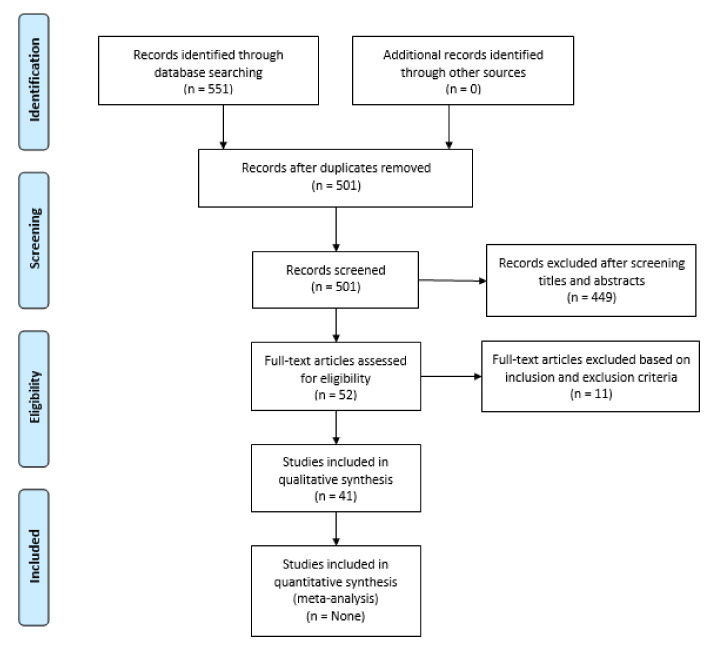
PRISMA flow diagram depicting the flow of information through the different phases of a systematic review.

The literature search retrieved a total of 551 articles. After applying the exclusion criteria and removing the duplicated papers, a total of 41 articles were included in our review. Figure 1 shows the PRISMA flow chart showing the selection process. Table 1 shows the summarized characteristics of each study that was included. There were seven case series [10,13,14,15,16,48,49,50], 23 case reports [18,19,20,21,22,23,24,25,28,29,30,31,32,34,35,36,37,38,40,41,42,43,52], and the rest were letters to the editor, brief reports, and a scientific letter. Two studies were international [15,16], twenty studies were from the USA [13,17,18,19,20,24,28,29,30,32,33,35,37,38,39,41,47,48,50,52], four from Brazil [31,43,44,51], three from United Kingdom [10,25,49], two from Italy [35,45], two from Spain [23,34], two from Iran [21,22], and one each from Mexico [14], Senegal [26], Germany [27], Turkey [40], Saudi Arabia [42], and France [45] (Table 1).

### 3.2. Patient Background 

The results of 159 patients were included in this review (Table 1). Sixty-three patients were female, 74 were male. Some studies did not report the sex of the patients (22 patients). The age of the children ranged from 24-h to 17-years old. From the 159 patients, 38 were reported with some type of cerebrovascular disease [12,13,14,15,16,17,18,19,20,21,22], 32 with different types of encephalitis [13,14,15,26,27,28,29,30,31,32], 22 with encephalopathy [10,13,24,25], and 10 patients with Guillain–Barre syndrome [13,14,42,43,44]. Sixty-five out of 159 patients with severe neurological manifestations, were reported with Multisystem Inflammatory Syndrome (MIS-C) [10,12,14,15,16,17,23,32,45,50,52,53,54] (Table 1). 

### 3.3. Central Nervous System Manifestations

#### 3.3.1. Cerebrovascular Disease

In this subset of pediatric SARS-CoV-2 patients with neurological findings, the most common manifestation was cerebrovascular disease (CVD), which was reported in 38 cases and included ischemic and hemorrhagic strokes, venous thrombosis, and cerebral arteriopathy. 

LaRovere et al., 2021 [13], in their case series in the USA, reported 12 cases with acute ischemic or hemorrhagic stroke. Eight patients with this diagnosis had underlying risk factors, including five patients that experienced a stroke during ECMO. Two cases were related to possible COVID-19-linked exacerbation of their primary neurological disorders (e.g., rupture of arteriovenous malformation rupture and a patient with moyamoya syndrome who had an ischemic stroke). Sánchez-Morales et al., 2021 [14] published two cases of acute ischemic stroke in a case series in Mexico. Both cases presented with underlying conditions: a 12-year-old male who had a previously undiagnosed aortic coarctation with severe hypertension in the upper extremities; and a 16-year-old female who had a history of M2 acute myeloid leukemia. The location of the ischemic strokes was reported in the left middle cerebral artery territory and the watershed areas of the brain, respectively. Lindan et al., 2020 [15] in a multinational study of neuroimaging manifestations in children with SARS-CoV-2 infection reported seven cases of stroke, characterized as thromboembolic or vasculitic. Finally, Beslow et al., 2020, [16] in an international case series, reported eight pediatric patients with strokes. Seven cases had an acute ischemic stroke, including one neonate. The other case was a nine-year-old boy with a cerebral sinovenous thrombosis of the right transverse sinus and right sigmoid sinus. 

Several case reports have been published about CVD in children with SARS-CoV-2. Schupper et al., 2020 [17] reported two cases with cerebrovascular symptoms. Both cases were diagnosed with MIS-C. A five-year-old boy presented with fever, cough, and pain and progressed to cardiogenic shock. Extracorporeal membrane oxygenation (ECMO) was necessary, and after five days, the patient developed a fixed and dilated right pupil. Imaging confirmed a right middle cerebral artery (MCA) infarction, cerebral edema, and diffuse contralateral subarachnoid hemorrhage (SAH). This patient was subsequently declared brain dead. The second case was a two-month-old boy who presented with respiratory failure and was found to have SARS-CoV-2 antibodies. After being placed on ECMO for eight days, this patient developed nonconvulsive status epilepticus. Imaging confirmed bilateral MCA and posterior cerebral artery (PCA) infarction with hemorrhagic transformation. 

Four other cases were reported in the United States of America by three authors. Kihira et al., 2020 [18] reported a fatal case of a five-year-old boy previously healthy, who presented with a three-day history of fever, cough, and abdominal pain. D-dimer at presentation was elevated. The patient progressed to cardiogenic shock and underwent ECMO cannulation. Four days later, his right pupil became dilated and non-reactive to light. Imaging revealed a large acute right anterior cerebral artery (ACA) and MCA territory infarction and left hemispheric subarachnoid hemorrhage. The patient was declared brain dead after three days. Appavu et al., 2020 [19] reported two cases of arterial ischemic strokes in previously healthy children within three to four weeks of COVID-19. The first case was an eight-year-old girl who presented with new-onset right hemiplegia and language impairment. Imaging revealed bilateral infarctions of the MCA territories. Magnetic resonance vessel wall imaging showed concentric mural enhancement of the left internal carotid artery consistent with inflammatory intracranial arteriopathy. The second case was a 16-year-old boy who presented with right hemiparesis and global aphasia. Brain MRI and MRA revealed a complete left MCA territory infarction, with irregularity of left M1-segment suggestive of arteritis. Gulko et al., 2020 [20] reported a 13-year-old girl who presented with headache, speech difficulties, and right hemiparesis. Imaging demonstrated acute-subacute infarcts in the left middle cerebral artery vascular territory. MRA of the brain revealed moderate focal stenosis within the M1 segment of the left MCA, and vessel wall imaging targeting the affected M1 segment demonstrated wall thickening and marked, concentric contrast enhancement at the site of the stenosis. The patient improved clinically and was discharged asymptomatic. 

Two cases of cerebrovascular disease were reported in Iran by Basirjafari et al., 2020 [21] and Mirzaee et al., 2020 [22]. The first author reported a 9-year-old boy who presented with cardiopulmonary arrest. The patient developed fixed and dilated pupils on the second day, and a head CT scan revealed brain edema and SAH occupying the basal cisterns, the interhemispheric cistern, and the Silvian fissures bilaterally. The patient died after two days of hospitalization. The authors ruled out cerebral aneurysms and parenchymal lesions. The second author reported a 12-year-old boy who presented with new-onset generalized seizures, rapidly progressing to right hemiparesis, and dysarthria, without respiratory symptoms. This case had a SARS-CoV-2 viral nucleic acid test positive in the nasopharyngeal swab and the cerebrospinal fluid (CSF). Imaging showed an acute infarction of the left MCA vascular territory, with irregular narrowing and banding of the proximal M1 segment, which could correspond to a focal cerebral arteriopathy. 

One case was reported with cerebral sinovenous thrombosis in Spain [23]. A 13-year-old girl presented to the emergency department (ED) with impaired consciousness and severe headache. The patient had a one-week history of fever, cough, odynophagia, and vomiting. Imaging of the brain revealed a right occipital intracerebral hemorrhage. Magnetic resonance angiogram (MRA) showed bilateral transverse sinus thrombosis with extension to the right sigmoid sinus and internal jugular vein. Laboratory studies found increased lactate dehydrogenase (LDH), serum ferritin, and C-reactive protein (CRP). Despite progressive clinical improvement with treatment, on the fifth day, the patient exhibited thrombosis progression to the posterior half of the superior sagittal sinus, bilateral femoral and iliac veins thrombosis, bilateral pulmonary thromboembolism. After adequate management, the child was discharged after 24 days, asymptomatic and without neurological sequela. 

#### 3.3.2. Encephalopathy, Encephalitis, and Encephalomyelitis 

LaRovere et al., 2021 [13] reported 15 cases with severe encephalopathy. Most of the cases were males (11 cases), and only one case had an underlying neurological condition. Five children presented brain MRI findings of white matter hyperintensities and splenial lesions. Eight out of fifteen patients were diagnosed with MIS-C. Four children died. In the same case series, eight children were reported with acute central nervous system (CNS) infection or acute disseminated encephalomyelitis (ADEM) without reporting fatalities in this subgroup. 

Three cases of encephalopathy were reported in the United States of America and the United Kingdom by Abel et al., 2020 [24] and Vraka et al., 2021 [25] respectively. The first case was reported in New York, a 33-month-old boy who met the criteria for MIS-C and developed a reversible encephalopathy. The child presented to the emergency department with a two-day history of fever, emesis, and rash. SARS-CoV-2 antibody test was positive. The patient presented pericardial effusion and sinus tachycardia. Supportive therapy for MIS-C was used. After six days at the hospital, the child became somnolent, hypotonic, and significantly weak. Head CT scan without contrast was unremarkable. EEG showed moderate background slowing. Lumbar puncture was negative for SARS-CoV-2. The following day the brain MRI revealed restricted diffusion in the bilateral lateral thalamic nuclei without T2 FLAIR recovery. Subsequently, the neurological status of the patient improved. EEG on day 12 showed mild diffuse slowing, and brain MRI on day 15th showed complete resolution of the thalamic lesions. The second author published two cases, a previously healthy 13-month-old girl who presented with altered mental status, seizures, and a three-day history of fever. Initial examination revealed decorticate posturing and a Glasgow Coma Score of five. She required intubation and management in the intensive care unit. EEG showed diffuse slow-wave background activity without epileptiform discharges. Imaging of the brain showed splenial lesions. The patient was treated with steroids and had a good evolution. The other case was a 10-year-old girl who presented with vomiting, lethargy, and a two-day history of pyrexia. After nine days of admission, the patient developed fluctuating sensorium and urinary incontinence, progressing to hypertonia, brisk reflexes, and right-sided Babinski sign. Brain imaging was unremarkable initially. After a good evolution, the patient was extubated and finally discharged. However, due to persistent right-arm neglect, an MRI at day 50 after the illness onset showed asymmetric bilateral high-signal lesions of the basal ganglia and the subcortical white matter in the frontal and temporal lobes. 

Encephalitis has been reported in several countries. In Senegal, Kahwagi et al., 2020, [26] reported a seven-year-old female who was diagnosed with COVID-19 after presenting cough, fever, and headache. On day six after admission, the patient presented several episodes of generalized tonic-clonic seizures that resolved with medication. After 11 days at the hospital, the patient was discharged with a negative brain MRI and negative nasopharyngeal swab for SARS-CoV-2. Three days later, the patient presented gait and behavior disturbances with osteotendinous hyperreflexia. EEG showed overall slowing of the pattern with the presence of pseudoperiodic complexes predominating in the frontotemporal area. The patient recovered after two months. In Germany, Lorenz et al., 2020, reported [27] a 24-h female newborn who developed therapy refractory fever and encephalitic symptoms 24 h after birth. The clinical picture progressed to lethargy and hyperexcitability at 54 h of life. The mother had symptoms of COVID-19 during the delivery. Both mother and newborn tested positive for SARS-CoV-2. CSF of the newborn tested negative for SARS-CoV-2. The patient developed respiratory distress managed with continuous positive airway pressure- and oxygen therapy. Viral pneumonia was confirmed in the chest radiograph. The patient was discharged 14 days after birth without symptoms. In the United States of America, two case reports were published by McAbee et al., 2020 [28] and Bhavsar et al., [29]. The first author published a case of an 11-year-old boy who presented with status epilepticus requiring multi-drug therapy. CSF demonstrated evidence of encephalitis. Electroencephalogram (EEG) revealed frontal intermittent delta activity. The head CT scan was normal. Complete recovery after six days was reported. The second author reported a 16-year-old boy who presented to the ED with fever, followed by generalized weakness and somnolence for two days. The neurological examination showed a patient difficult to arouse, confused, and with incoherent speech. He also presented episodes of focal seizures successfully treated. Protein concentration in the CSF was increased, with mononuclear pleocytosis and hypoglycorrhachia. Meningoencephalitis PCR panel in the CSF was negative and SARS-CoV-2 was not detected. EEG showed slow background without epileptiform discharges or seizures. The patient was managed and eventually improved. He was discharged on day 15.

Two cases of anti-N-Methyl-D-Aspartate receptor (NMDAR) encephalitis have been reported in the literature. Sánchez-Morales et al., 2021 [14] in Mexico reported a 14-year-old boy previously healthy who presented altered behavior and mental status, seizures, insomnia, and orolingual dyskinesias. Antibodies to both NMDAR and SARS-CoV-2 were detected in the CSF. SARS-CoV-2 PCR test in CSF was positive as well. After treatment with steroids and immunoglobulin, the patient had partial recovery. The second case was reported in the United States of America by Burr et al., 2021 [30]. This case was a 23-month-old girl who presented to the emergency department with dehydration and fever. Two days after admission, she presented several seizures. SARS-CoV-2 PCR from the CSF was negative. MRI of the brain was normal. The patient had a resolution of the fever two weeks later but had worsening encephalopathy. NMDAR-IgG antibody was positive in serum and CSF. Over the following weeks, the symptoms resolved. 

Acute disseminated encephalomyelitis (ADEM) has been reported in two publications. In Brazil, De Miranda Henriques-Souza et al., 2020 [31] reported a 12-year-old girl who presented with skin rash, headache, and fever. Five days after the onset of the symptoms, the patient presented acute, progressive, and symmetrical muscle weakness and paresthesia in the lower limbs without sphincter dysfunction. The neurological symptoms worsened with abolition of oculocephalic, corneal, cough, and gag reflexes. MRI of the brain on the seventh day showed extensive bilateral and symmetric restricted diffusion involving the subcortical and deep white matter and a focal hyperintense T2/fluid-attenuated inversion recovery (FLAIR) lesion in the corpus callosum, with restricted diffusion as well. Cervical spine MRI revealed longitudinally extensive cervical myelopathy, affecting both white and gray matter. Mechanical ventilation was required, and steroid therapy was used. The patient had partial recovery of the neurological symptoms after 60 days. In the United States of America, McLendon et al. 2021, [32] reported a 17-month-old girl who started with fatigue and progressively worsening weakness and unsteady gait. Her neurological examination showed multiple neurological focal symptoms, including rigidity of the left upper extremity and paresis of the right upper extremity. The physical exam also revealed hyperreflexia in the lower extremities, with truncal ataxia and gait instability. MRI of the brain revealed multifocal hyperintense T2 FLAIR signals in the bilateral subcortical and periventricular white matter without contrast enhancement. MRI of the spine without contrast was unremarkable. EEG showed diffuse slowing. Steroid therapy was used to manage the patient. The patient’s neurological symptoms improved totally after two months of post-discharge follow-up.

#### 3.3.3. Acute Fulminant Cerebral Edema 

Five cases have been reported in the literature with acute cerebral edema in children with SARS-CoV-2 infection. This complication has had high mortality. Kim et al., 2020, [33] reported a seven-year-old boy with no significant past medical history presented with a three-day history of fever, headaches, abdominal pain, and emesis. Two days later, the patient developed severe neck pain and headache. The child progressed to rapid neurological deterioration, becoming unresponsive with left gaze deviation. Initial head CT scan and EEG were negative. During the next hours, the EEG developed intermittent polymorphic delta activity in the right temporal region. A new CT scan of the head revealed diffuse cerebral edema. Laboratory results were consistent with severe inflammation. Ultimately the patient was diagnosed with brain death. The other four cases were reported by LaRovere et al., 2021 [13]. Two out of four cases were diagnosed with MIS-C, and only one child survived. 

#### 3.3.4. Seizures, Status Epilepticus 

Seizures were reported in five patients as the primary neurological manifestation of children with SARS-CoV-2 infection. Three of those cases were self-limited seizures in afebrile and previously healthy children. Garcia-Howard et al., 2020 [34] reported a three-month-old girl with COVID-19 infection who presented five episodes of afebrile focal motor seizures. EEG and MRI of the brain were normal. She had a favorable response to medical treatment. It is of note that the infant and her mother carried a loss-of-function variant in the PRRT2 gene, which is associated with benign familial infantile convulsions. Bhatta et al., 2020 [35] presented a case of a new-onset afebrile seizure in an 11-year-old male child. The patient presented two episodes of generalized tonic-clonic seizures without any other symptoms. During follow-up, the child was asymptomatic. Finally, in Italy, Silvagni et al., 2020 [36] reported a four-year-old girl who was admitted to the pediatric ED for the nocturnal onset of prolonged afebrile seizures at home. The patient did not have any history of fever or respiratory symptoms. Her neurological function rapidly improved with adequate treatment. The labs were normal except for a positive nasopharyngeal swab test for SARS-CoV-2 that was positive. Neurologic exam and video-EEG recording corresponded to possibly self-limiting focal epilepsy with temporo-occipital spikes. Her clinical condition improved, and she was discharged under a local surveillance system for COVID-19 and follow-up. 

Two cases were reported in the USA with status epilepticus. Farley et al. 2020, [37] reported an eight-year-old boy who presented to the ED with a left-sided focal seizure. The episode lasted for more than 30 min, and it was treated with lorazepam. Due to respiratory deterioration, the child was intubated, and a SARS-CoV-2 test was performed with a positive result. He was afebrile and had no respiratory symptoms before the event. The child had a good clinical evolution and was extubated very soon. The 24-h EEG indicated a diffuse cerebral dysfunction of non-specific etiology. He was discharged home to quarantine and neurologic follow-up. The other case was reported by Chegondi et al., 2020, [38], a two-year-old girl who was admitted to the ED with fever and generalized tonic-clonic seizures that were treated with lorazepam and levetiracetam. The patient progressed to respiratory depression and was intubated and transferred to a Pediatric Intensive Care Unit (PICU). COVID-19 test was positive. CRP and LDH were both elevated. The 24-h cEEG showed generalized slowing without recurrence of seizures. Her parents reported she never had nasal congestion, cough, or gastrointestinal symptoms. CSF was normal. She remained afebrile during her PICU stay and had a good clinical evolution. 

#### 3.3.5. Acute Myelitis

The first presentation of acute myelitis in a pediatric SARS-CoV-2-positive patient was reported in New Mexico, in the United States of America, by Kaur et al., 2020 [39]. A three-year-old girl, previously healthy, presented progressive muscle weakness and decreased sensation. The patient evolved to flaccid quadriparesis and respiratory failure requiring intubation. The neurologic exam showed the absence of cough and gag reflexes, with all other cranial nerves intact, flaccid quadriparesis with areflexia, and no response to pain below the neck. Initial MRI of the cervical spine revealed T2-hyperintense edema involving most of the transverse aspect of the spinal cord from the lower medulla to midthoracic level. CSF showed mild pleocytosis. SARS-CoV-2 test was positive in the nasopharyngeal swab. CSF was negative for SARS-CoV-2 by PCR. The patient was diagnosed with longitudinally extensive transverse myelitis (LETM) and was treated with intravenous steroids, immunoglobulin, and therapeutic plasma exchange. The girl never had respiratory symptoms. At the moment of the report, the patient was receiving rituximab and the follow-up images were showing interval reduction of the lesion and decreased edema of the upper cervical cord and the medulla. 

Another case was reported in Turkey by Guler et al., 2020 [40], a 14-year-old girl who presented with a sudden-onset loss of right arm and leg strength. She tested positive for SARS-CoV-2 in the nasopharyngeal swab. MRI of the spinal cord showed a contrast-enhancing lesion causing expansion at the C2–C5 level. Transverse myelitis was suspected, and treatment with intravenous immunoglobulin and steroids was started. Fentanyl and gabapentin were also used to treat neuropathic pain in the patient’s right arm and leg. After five days of steroid treatment, the patient showed partial neurological improvement. The patient was discharged 16 days after admission. 

#### 3.3.6. Other Neurological Manifestations 

A case of an acute event was reported by Dugue et al., 2020 [41]. A six-week-old term male infant presented with a one-day history of cough, fever, and brief episodes of sustained upward gaze associated with bilateral leg stiffening. The event was not associated with feeding and lasted 10 s. EEG monitoring showed an excess of sharp temporal transients for age and intermittent vertex delta slowing with normal sleep-wake cycling. A nasal swab was positive for SARS-CoV-2. The patient was discharged after one day. 

### 3.4. Peripheral Nervous System Manifestations

#### 3.4.1. Guillain–Barre Syndrome

Ten cases have been reported of Guillain-Barre (GBS) in SARS-CoV-2 pediatric patients. Eight cases were male, and there was no fatality reported. LaRovere et al., 2021 [13] reported four cases with GBS. Two patients were of school age, and two patients were teenagers. Only one case was diagnosed with MIS-C in this subset of cases, and 75% of the children were previously healthy. Three additional cases were reported in Mexico by Sánchez-Morales et al., 2021 [14]. The cases were in the range of ages between 9 and 14 years old. Two were recurring cases, as they were diagnosed with GBS before. The three cases showed a pattern of acute inflammatory demyelinating polyneuropathy (ADIP) in the nerve conduction studies. Sánchez-Morales et al., 2021 [14] described the outcome of the patients with the Hughes scale, which was normal (0) in the three cases. 

Three case reports have been published, one in Saudi Arabia [42] and two in Brazil [43,44]. Khalifa et al., 2020 [42] reported one case of an 11-year-old boy who presented in the ED with acute onset of unsteady gait, inability to climb stairs, and tingling sensation in the lower extremities. The patient had a history of upper respiratory tract infection three weeks before. Initial neurological examination showed symmetrical muscle weakness and hypotonia in the lower limbs, as well as the abolition of the ankle and knee osteotendinous reflexes. MRI of the spine showed postcontrast enhancement of the cauda equina nerve roots. Nerve conduction velocity studies revealed a pattern consistent with demyelinating polyneuropathy. CSF confirmed albumin cytological dissociation. The patient was found positive for SARS-CoV-2 in a nasal swab test. Other causes of GBS were ruled out. The patient showed gradual improvement of the motor strength and was discharged home. At the time of the report, a follow-up nerve conduction study was showing improvement of the parameters. 

In Brazil, Frank et al., 2020 [43] reported a 15-year-old boy who presented with weakness and pain in the lower limbs that ascended to the upper limbs. The patient had a history of headache, fever, and sweating before the onset of these symptoms. MRI of the lumbar spine was normal. Electroneurography showed abnormalities consistent with the acute motor axonal neuropathy variant of GBS. At the time of the report, the patient was undergoing motor physiotherapy to recover the persistent muscle weakness in the four extremities. The other case reported in Brazil was published by Araújo et al., 2021 [44], a 17-year-old girl who presented to the emergency with a 48-h history of low back pain, followed by weakness of the extremities. Eight days earlier, the patient presented fever, abdominal pain, and diarrhea. The neurologic exam showed symmetrical flaccid tetraparesis worse in the lower limbs. CSF analysis exhibited albumin cytological dissociation. Electroneuromyography revealed a pattern of demyelinating polyradiculoneuropathy. Remarkable in this case is that SARS-CoV-2 was detected on RT-PCR assay in the CSF. Magnetic resonance imaging of the neuroaxis showed cervical and cauda equina nerve roots enhancement, a sign of radiculitis. The patient was treated with intravenous immunoglobulin with motor improvement. The child was discharged with a GBS disability score of 3. 

#### 3.4.2. Cranial Neuropathy

A case of cranial polyneuropathy in a pediatric patient with COVID-19 was reported in France by Roussel et al., 2020 [45]. The case was a six-year-old girl with a history of sickle cell anemia complicated with cerebral vasculopathy. The patient underwent a hematopoietic stem cell transplantation. Twenty-one days after the procedure, she started with bilateral facial palsy, left trigeminal hypoesthesia, and voice alterations. Additionally, one day later, the girl began to suffer swallowing impairment. MRI of the brain revealed T2-FLAIR hypersignals of both facial nerves, in both intracranial and meatal segments, as well as the left hypoglossal nerve. Four days after the onset of the neurological symptoms, the patient developed respiratory symptoms and fever. The nasopharyngeal swab was positive for SARS-CoV-2. The patient required mechanical ventilation. The patient started to improve clinically around day 60. 

Lonardi et al., 2021 [45] in Italy, reported a case with a unilateral third cranial nerve palsy in a two-year-old child. The patient has a past medical history of sphingosine phosphate lyase insufficiency syndrome. The cranial nerve paralysis started three weeks after the resolution of SARS-CoV-2 infection, which was complicated with MIS-C. The child presented with right third cranial nerve palsy, with exotropia, ptosis, and mydriasis. Investigations revealed elevated cerebrospinal fluid/serum quotient of albumin. The patient was treated with an oral steroid. 

A Bell’s palsy in a child with SARS-CoV-2 was reported by Theophanous et al., 2021 [46]. The case was a six-year-old boy with a history of prematurity (born at 30 weeks), hyper IgM syndrome, gastrostomy tube feeding, etc., who presented to the pediatric ED with a one-day history of right-sided facial droop. Neurologic examination showed a right-sided facial palsy with the inability to close the right eye and right-sided mouth droop with drooling, a House-Brackmann grade IV. SARS-CoV-2 test was positive in the nasopharyngeal swab. The patient was treated with steroid and acyclovir with improvement of the symptoms. 

Two cases of left optic neuritis were reported by Sánchez-Morales et al., 2021 [13]. One case was a 15-year-old girl with bilateral optic neuritis and a sixth cranial nerve paresis. The other case was a 14-year-old girl with left optic neuritis. Both cases were previously healthy. The girls recovered their visual acuity completely, and there was no evidence of other demyelinating diseases. 

### 3.5. Multisystem Inflammatory Syndrome in Children (MIS-C) and Neurological Complications 

Feldstein et al., 2020 [48] in a multicenter study across the United States of America, in 186 children diagnosed with MIS-C, reported 10 cases with severe neurological complications, including encephalitis, aseptic meningitis, demyelinating disorders, seizures, and coma. In the United Kingdom, Abdel-Mannan et al., 2020 [10] reported neurological symptoms in four out of 27 children diagnosed with MIS-C. The symptoms included encephalopathy, headache, brainstem signs with dysarthria and dysphagia, meningism, and cerebellar ataxia. 

Two studies in New York among pediatric patients diagnosed with MIS-C were published by Dufort et al., 2020 [49] and Cheung et al., 2020 [50]. The former study reported headache, altered mental status, or confusion in 30 cases out of 99 children diagnosed with MIS-C. The latter study reported headache, stiff neck, and vision change in 8 out of 17 children with MIS-C. 

In the case series published by LaRovere et al., 2021 [13] 126 out of 365 patients with neurological involvement met the criteria for MIS-C, which represents 35%. Forty-three out of 365 patients presented life-threatening neurological involvement, and 20 of these children met the criteria for MIS-C. 

A case report relating to MIS-C and neurological involvement was published by DePaulis et al., 2020 [51]. The case was a four-year-old girl, previously healthy, who presented with fever, vomiting, and skin rash. The patient became lethargic on day three and started complaining of severe myalgia. Due to clinical deterioration, the patient was intubated. Inflammatory markers were elevated. CSF showed pleocytosis and increased proteins. The patient was treated with antibiotics, acyclovir, dobutamine (for 16 h), and intravenous immunoglobulin. She was extubated on day 8 and finally discharged, fully recovered, on day 17. 

Verkuil et al., 2020 [52], in Philadelphia, reported a case of secondary pseudotumor cerebri in a 14-year-old girl. The patient was previously healthy, with a body mass index of 19.2 kg/m^2^. She presented to the hospital with a five-day history of fever, headache, rash, diarrhea, and dyspnea. The patient progressed to respiratory failure and septic shock requiring mechanical ventilation. An echocardiogram detected diffuse dilation of the right coronary artery. She was treated with intravenous immunoglobulin and steroids. After extubation on day six, she presented an esotropia. Examination showed right abducens nerve palsy. Bilateral papilledema was found in the dilated fundus examination, with left-disc hemorrhages. Magnetic resonance venogram was consistent with increased intracranial pressure, and lumbar puncture revealed elevated opening pressure. The patient was treated with acetazolamide and prednisone. After a two-month follow-up, the patient had complete resolution of the papilledema, left-disc hemorrhage, and sixth cranial nerve palsy.

## 4. Discussion

During the last 20 years, the world has experienced three epidemics linked to coronaviruses: severe acute respiratory syndrome (SARS) in 2002, the Middle East respiratory syndrome (MERS) in 2012, and the ongoing pandemic of severe acute respiratory syndrome coronavirus 2 (SARS-CoV-2) [53]. The novel SARS-CoV-2 causes a clinical syndrome with pulmonary manifestations that have been well characterized [54]. However, there are reports of neurological manifestations in patients with SARS-CoV-2 infection in both adult and pediatric populations. 

COVID-19 usually courses with mild symptoms in children; however, serious complications may occur during both acute infection and the multisystem inflammatory syndrome in children (MIS-C) [55]. The neurological manifestations related to the central nervous system elucidated in the present review include strokes, encephalopathy, encephalitis, seizures, acute cerebral edema, acute transverse myelitis, acute myelitis, and cerebellar ataxia. These symptoms could be produced by direct neuroinvasion of the SARS-CoV-2 and/or indirectly with the activation of the immune system. 

Several routes for possible viral neuroinvasion have been proposed, including transsynaptic spread via the olfactory nerve, infection of the endovascular endothelium, or leukocyte migration across the blood–brain barrier (BBB) [54]. The entry of the SARS-CoV-2 into the human cells is thought to be mediated by the interaction between the ACE2 receptors of the cells and the spike proteins of the SARS-CoV-2. ACE2 receptor expression has been reported in the cerebrum, cerebellum, brainstem, retina, and olfactory mucosa. The neurons, vascular pericytes, smooth muscle cells, and glia express the ACE2 receptor. Other possible receptors associated with the entry of the virus are basigin (BSG; CD147), neuropilin-1 (NRP1), transmembrane serine protease 2 and 4 (TMPRSS2/4), and cathepsin L (CTSL) [56]. One of the hypotheses explaining milder clinical presentations of COVID-19 in children is the lower expression of entry receptors of the virus in the airway epithelium in children compared to adults [57,58,59]. 

SARS-CoV-2 could also indirectly damage the central nervous system via activation of the immune response, damaging the neuronal tissue [60]. SARS-CoV-2 affects the CNS via systemic and local inflammatory response causing cytokines storming and immune cell reactivation [61]. The rarity with which the virus has been found in the CSF in patients with clinical evidence of brain inflammation linked to SARS-CoV-2 implies that immune-mediated damage is more important than viral replication in neurons [62]. 

Stroke and encephalopathy were the predominant neurological syndromes associated with SARS-CoV-2 infection in children in this review. Hypercoagulable states indicated by elevated D-dimer, prolongation of prothrombin time (PT), activated partial thromboplastin time (aPTT), and thrombocytopenia have been observed with the SARS-CoV-2 infection [63]. The interaction between the virus and the ACE2 receptors expressed on vascular endothelial cells may trigger a pro-inflammatory response and a pro-coagulable state by initiating vasculitis and disruption of vascular integrity, with subsequent activation of the clotting cascade [55]. The antiphospholipid syndrome may also play a role in the thrombotic events linked to SARS-CoV-2 infection, which has been reported in adults [64]. Other hypotheses include the overactivation of the complement system and the effect of the neutrophil extracellular traps [65]. 

Encephalopathy refers to a clinical state of altered mental status, manifesting as confusion, disorientation, behavioral changes, or other cognitive impairments, with or without inflammation of the brain tissue [66]. Encephalitis is characterized by the inflammation of the brain parenchyma associated with neurological dysfunction [67]. The inflammation of the brain can be produced by direct infection, post-infectious processes such as acute disseminated encephalomyelitis (ADEM), or non-infectious condition such as N-methyl-D-aspartate receptor (NMDAR) encephalitis [66]. 

ADEM, a form of autoimmune encephalitis, is an immune-mediated, inflammatory demyelinating disease of the central nervous system that can affect children and young adults after infections or immunizations [68] and was reported in two cases [31,32] in the present review. The mean age of onset is between 3.6 and 7 years, without differences in sex [69]. In the pathogenesis, the molecular mimicry between microbial epitopes and myelin antigens such as myelin basic protein (MBP), proteolipid protein (PLP), and myelin oligodendrocyte glycoprotein (MOG) is considered the most important mechanism of the immune-mediated injury [68,70]. 

Two reports of anti-NMDA receptor encephalitis associated with SARS-CoV-2 infection in children were found [14,30]. In a previous multi-institutional observational study, children accounted for 37% of the patients with this type of encephalitis [71]. The most common symptoms were dyskinesia, personality change, seizures, and cognitive disorders [72]. This type of encephalitis has also been reported in adults [73,74]. 

Guillain–Barre syndrome was found in 10 patients. It is characterized by acute, ascending, areflexic paralysis with albuminocytologic dissociation [75]. Molecular mimicry between a microbial and neural antigen is an essential driving force in this disease [76]. 

In the present review, 38% of the cases reported with neurologic manifestations were diagnosed with MIS-C. Neurological symptoms associated with multisystem inflammatory syndrome in children (MIS-C) were first described by Dufort et al., 2020 [49]. MRI findings related to MIS-C were described by Abdel-Mannan et al., 2020, in four children [10]. The pathophysiology of the neurologic complications in MIS-C remains unclear [77]. A multisystem inflammatory syndrome in adults has also been reported [78]. 

### Limitations

Most of the studies mentioned are case reports, which limits the interpretation and generalization of the results. Furthermore, cases will continue to accumulate, given that the pandemic is ongoing. The ethnicity of the patients was not included in the study which may have an impact in the outcomes of the patients. Further studies are needed to elucidate the role of the ethnicity in the COVID-19 impact. 

## 5. Conclusions

In conclusion, we aimed to review the current literature on pediatric patients with coexisting severe neurological manifestations and SARS-CoV-2 infections. Though severe neurological symptoms are not typical manifestations of COVID-19 or MIS-C, this review compiles the findings of a number of cases with life-threatening neurological disorders. Given the prevalence of neurologic symptoms in children infected with SARS-CoV-2, healthcare providers should consider SARS-CoV-2 testing in pediatric patients who present with neurologic presentations, even when only neurologic symptoms are seen. In addition, many of these patients were unaware of COVID-19 exposure and positivity upon arrival to the hospital, so testing is warranted to prevent infectious spread.

## Figures and Tables

**Table 1:** Characteristics of the studies included in the review.

Study	Type of Study	Country	Journal	Patients with Severe Neurological Manifestations, Sex, Age (Years)	Severe Neurological Manifestations Multisystem Inflammatory Syndrome in Children (MIS-C)
LaRovere et al., 2021 [13]	Case series	United States of America	JAMA Neurology	*n =* 43 Male: 27 Female: 16 12 (7–15)	Acute ischemic/hemorrhagic stroke: 12 Severe Encephalopathy: 15 Acute Central Nervous System infection/Acute Disseminated Encephalomyelitis: 8 Acute fulminant cerebral edema: 4 Guillain-Barre syndrome: 4 MIS-C: 20
Sánchez-Morales et al., 2021 [14]	Case series	Mexico	Child’s Nervous System	*n* = 10 Male: 5 Female: 5 Age: 2–16	Acute Ischemic Stroke: 2 Acute Cerebellar Ataxia: 1 Anti-N-Methyl-D-Aspartate Receptor Encephalitis: 1 Guillain-Barre Syndrome: 3 Optic Neuritis: 2 Myositis with Rhabdomyolysis: 1
Lindan et al., 2020 [15]	Case series	International	The Lancet Child & Adolescent Health	*n* = 38 Male: 20 Female: 18 Age: 0.17–16	Neuroimaging manifestations: ADEM-like changes: 16 Myelitis: 8 Thrombotic/vasculitic: 7 Neural enhancement: 13 MIS-C: 11
Beslow et al., 2020 [16]	Case series	International	Annals of Neurology	*n* = 8 Male: 5 Female: 3 Age: 0.01–16	Neonatal Acute Ischemic Stroke: 1 Childhood Acute Ischemic Stroke: 6 Childhood Cerebral Sinovenous Thrombosis: 1 MIS-C: 2
Schupper et al., 2020 [17]	Letter to the Editor	United States of America	Child’s Nervous System	*n* = 2 5-year-old male 2-month-old male	Acute ischemic and hemorrhagic stroke: 1 Acute ischemic stroke with hemorrhagic transformation: 1 MIS-C: 2
Kihira et al., 2020 [18]	Case report	United States of America	Pediatric Radiology	*n* = 1 5-year-old male	Acute ischemic and hemorrhagic stroke MIS-C: 1
Appavu et al., 2021 [19]	Case report	United States of America	Pediatrics	*n* = 2 8-year-old female 16-year-old male	Case 1: Acute ischemic stroke Case 2: Acute ischemic stroke
Gulko et al., 2020 [20]	Case report	United States of America	American Journal of Neuroradiology	*n* = 1 13-year-old female	Acute ischemic stroke (focal cerebral arteriopathy)
Basirjafari et al., 2020 [21]	Case report	Iran	Journal of Medical Virology	*n* = 1 9-year-old male	Acute hemorrhagic stroke (subarachnoid hemorrhage)
Mirazee et al., 2020 [22]	Case report	Iran	Radiology	*n* = 1 12-year-old male	Acute ischemic stroke (evidence of focal cerebral arteriopathy)
Bastidas et al., 2020 [23]	Case report	Spain	Neurology Clinical Practice	*n* = 1 13-year-old female	Cerebral sinovenous thrombosis
Abel et al., 2020 [24]	Case report	United States of America	Neurology	*n* = 1 2-year-old male	Encephalopathy MIS-C: 1
Vraka et al., 2021 [25]	Case report	United Kingdom	Case Reports in Neurological Medicine	*n* = 2 13-month-old female 10-year-old female	Encephalopathy: 2
Kahwagi et al., 2021 [26]	Letter to the Editor	Senegal	Revue Neurologique	*n* = 1 7-year-old female	Encephalitis
Lorenz et al., 2020 [27]	Letter to the Editor	Germany	JAMA Pediatrics	*n* = 1 24-h female newborn	Encephalitic syndrome (newborn)
McAbee et al., 2020 [28]	Case report	United States of America	Pediatric Neurology	*n* = 1 11-year-old child **	Encephalitis
Bhavsar et al., 2020 [29]	Case report	United States of America	Neurology Clinical Practice	*n* = 1 16-year-old male	Encephalitis
Burr et al., 2021 [30]	Clinical letter	United States of America	Pediatric Neurology	*n* = 1 23-month-old female	Anti-N-Methyl-D-Aspartate Receptor Encephalitis
de Miranda Henriques-Souza et al., 2021 [31]	Case report	Brazil	Neuroradiology	*n* = 1 12-year-old female	Acute disseminated encephalomyelitis
McLendon et al., 2021 [32]	Case report	United States of America	Pediatrics	*n* = 1 17-month-old female	Acute disseminated encephalomyelitis
Kim et al., 2020 [33]	Clinical letter	United States of America	Pediatric Neurology	*n* = 1 7-year-old male	Fatal cerebral edema MIS-C: 1
Garcia-Howard et al., 2020 [34]	Case report	Spain	Frontiers in Pediatrics	*n* = 1 3-month-old female	Benign infantile seizures
Bhatta et al., 2020 [35]	Case report	United States of America	Cureus	*n* = 1 11-year-old male	New-onset isolated afebrile seizure
Silvagni et al., 2020 [36]	Case report	Italy	Research Square	*n* = 1 4-year-old female	Self-limited focal epilepsy
Farley et al., 2020 [37]	Case report	United States of America	American Journal of Case Reports	*n* = 1 8-year-old male	Status epilepticus
Chegondi et al., 2020 [38]	Case report	United States of America	Cureus	*n* = 1 2-year-old female	Febrile status epilepticus
Kaur et al., 2020 [39]	Clinical letter	United States of America	Pediatric Neurology	*n* = 1 3-year-old female	Transverse Myelitis
Guler et al., 2020 [40]	Case report	Turkey	New Trends in Medicine Sciences	*n* = 1 14-year-old female	Transverse myelitis
Dugue et al., 2020 [41]	Case report	United States of America	Neurology	*n* = 1 6-week-old male	Acute event
Khalifa et al., 2020 [42]	Case report	Saudi Arabia	Journal of Pediatric Infectious Disease Society	*n* = 1 11-year-old male	Guillain–Barre syndrome
Frank et al., 2020 [43]	Case report	Brazil	Journal of Tropical Pediatrics	*n* = 1 15-year-old male	Guillain–Barre syndrome
Araújo et al., 2021 [44]	Brief report	Brazil	The Pediatric Infectious Disease Journal	*n* = 1 17-year-old female	Guillain–Barre syndrome
Roussel et al., 2020 [45]	Letter to the Editor	France	Pediatric Blood & Cancer	*n* = 1 6-year-old female	Cranial polyneuropathy (cranial nerves V, VII, and IX, XII)
Lonardi et al., 2021 [46]	Correspondence	Italy	Pediatric Neurology	*n* = 1 2-year-old **	Third cranial nerve palsy MIS-C: 1
Theophanous et al., 2020 [47]	Case report	United States of America	Brain & Development	*n* = 1 6-year-old male	Bell’s palsy
Feldstein et al., 2020 [48]	Case series	United States of America	New England Journal of Medicine	*n* = 10 * **	Encephalitis, aseptic meningitis, or demyelinating disorder: 4 Seizures: 3 Coma: 3 MIS-C: 10
Abdel-Mannan et al., 2020 [10]	Case series	United Kingdom	JAMA Neurology	*n* = 4 Male: 2 Female: 2 Age: 8–15	Encephalopathy: 4 Headache: 3 Dysarthria/dysphagia: 2 Meningism: 1 Cerebellar ataxia: 1 MIS-C: 4
Dufort et al., 2020 [49]	Case series	United Kingdom	New England Journal of Medicine	*n* = 2 * **	Altered mental status or confusion: 2 MIS-C: 2
Cheung et al., 2020 [50]	Case series	United States of America	New England Journal of Medicine	*n* = 8 * **	Headache, stiff neck, vision change MIS-C: 8
De Paulis et al., [51]	Brief report	Brazil	The Pediatric Infectious Disease Journal	*n* = 1 4-year-old female	Confusion, somnolence MIS-C: 1
Verkuil et al., 2020 [52]	Case report	United States of America	The Lancet	*n* = 1 14-year-old female	Pseudotumor cerebri MIS-C: 1

* Age was not included in the study. ** Sex was not included in the study.

## Data Availability

Not applicable.
